# Canine infection with *Dirofilaria immitis*, *Borrelia burgdorferi*, *Anaplasma* spp., and *Ehrlichia* spp. in the United States, 2013–2019

**DOI:** 10.1186/s13071-020-04514-3

**Published:** 2021-01-06

**Authors:** Susan Little, Jennifer Braff, Joshua Place, Jesse Buch, Bhagya Galkissa Dewage, Andrew Knupp, Melissa Beall

**Affiliations:** 1 Department of Veterinary Pathobiology, College of Veterinary Medicine, Oklahoma State University, Stillwater, OK USA; 2 IDEXX Laboratories, Inc., Westbrook, ME USA

**Keywords:** 4DxPlus, *Anaplasma*, Antibody, Antigen, *Borrelia burgdorferi*, Canine, *Dirofilaria immitis*, *Ehrlichia*

## Abstract

**Background:**

Dogs in the US are commonly infected with vector-borne pathogens, including heartworm and tick-borne disease agents. The geographic distribution of both arthropod vectors and the pathogens they transmit continues to expand.

**Methods:**

To describe the current geographic distribution and prevalence of antigen of *Dirofilaria immitis* and antibody to *Borrelia burgdorferi*, *Ehrlichia* spp., and *Anaplasma* spp. in dogs, we summarized over 144 million test results from 2013 to 2019, inclusive, by county, state, and region. Canine seroprevalence by state was compared to population-adjusted human reports of tick-borne diseases.

**Results:**

Results varied regionally, with *D. immitis* antigen and *Ehrlichia* spp. antibodies more frequently detected in the Southeast (2.6% and 5.2%, respectively) and antibody to *B. burgdorferi* and *Anaplasma* spp. most common in the Northeast (12.1% and 7.3%, respectively). Overall, percent positive test results to *D. immitis* decreased in the Southeast by 33.3% when compared to earlier summaries using the same strategy (from 3.9 to 2.6%). Geographic expansion of areas where dogs commonly test positive for *Ehrlichia* spp. was evident, likely because of a change in the test made in 2012 to allow detection of antibodies to *E. ewingii* concomitant with expansion of vector tick populations. Percent positive test results to *Ehrlichia* spp. increased in every region; this shift was particularly pronounced in the Southeast, where percent positive test results increased fourfold (from 1.3 to 5.2%). Continued geographic expansion of *B. burgdorferi* and *A. phagocytophilum* was apparent in the Northeast, Midwest, and Upper South, although canine seroprevalence of antibody to *B. burgdorferi* was much lower than prior surveys in many Lyme-endemic areas. Annual reports of human cases of Lyme disease, ehrlichiosis, and anaplasmosis were associated with percent positive canine results by state for the three tick-borne disease agents (*R*^2^ = 0.812, 0.521, and 0.546, respectively). Within endemic areas, percent positive test results for all three tick-borne agents demonstrated evidence of geographic expansion.

**Conclusions:**

Large scale analysis of results from screening dogs in practice for evidence of vector-borne infections, including those with zoonotic importance, continues to be a valuable strategy for understanding geographic trends in infection risk over time.
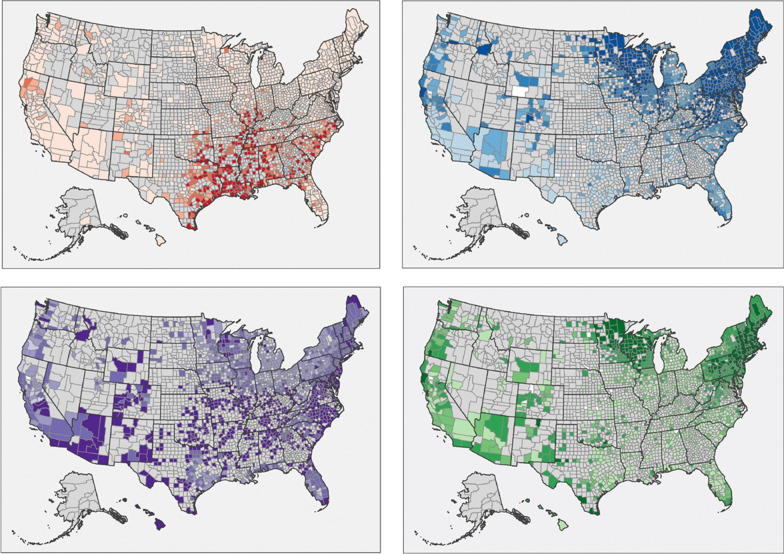

## Background

National summaries of canine vector-borne disease seroprevalence generated from testing individual dogs in practice aid understanding of pathogen distribution and provide insights into geographic and temporal changes [1, 2]. To facilitate early diagnosis, treatment, and prevention, advisory boards recommend that all dogs receiving veterinary care be evaluated annually for vector-borne infections [3]. Accordingly, each year in the US, millions of dogs are tested for antigen of heartworm (*Dirofilaria immitis*) and antibody to tick-borne disease agents, most commonly *Borrelia burgdorferi*, agent of Lyme borreliosis; *Anaplasma* spp., which cause anaplasmosis in people and animals; and *Ehrlichia* spp., causative agents of human and canine ehrlichiosis [1, 2, 4, 5]. In addition to benefiting canine patient health, analysis of the aggregated results captured using the same test method over many years can serve as a bellwether to identify areas where infection risk may be changing [6–8].

Despite the widespread availability of preventives, ticks are common on dogs across the USA, and evidence suggests the prevalence of heartworm infection and of antibodies to tick-borne disease agents is increasing in some regions [9–12]. These increases likely result from a combination of factors including increased vector populations, resulting in more intense transmission, geographic spread of natural maintenance cycles for infection, and translocation of infected dogs [11–16]. Canine serologic evidence of past or current tick-borne infection also correlates with human case reports on a county- and state-wide basis [2, 6, 10, 17], and the number of human cases is similarly increasing [18]. In the present article, we update our earlier publications by reporting the percent positive test results of dogs evaluated by veterinarians in the US from 2013 to 2019, documenting continued changes in both distribution of these infections and overall infection risk.

## Materials and methods

### Source of data

Results for the present analysis and summary (2013–2019) were generated using USDA-licensed test kits (IDEXX Laboratories, Inc.) and included: SNAP® 4Dx® Plus Test kit, an in-clinic enzyme-linked immunosorbent assay (ELISA) for detection of *D. immitis* antigen and canine antibodies to *B. burgdorferi*, *Ehrlichia* spp. (*E. canis*, *E. ewingii*), and *Anaplasma* spp. (*A. phagocytophilum* and *A. platys*); SNAP® HW RT Test kit, an in-clinic ELISA for the detection of *D. immitis* antigen in canine serum, plasma, or whole blood. In addition, results generated from microtiter plate ELISA tests for the detection of *D. immitis* antigen (e.g., PetChek® Heartworm PF Test) and canine antibodies to *B. burgdorferi*, *Ehrlichia* spp. (*E. canis*, *E. ewingii*), and *Anaplasma* spp. (*A. phagocytophilum* and *A. platys*) in canine serum or plasma at a reference laboratory were included. The performance of each test and associated diagnostic reagents has been reported previously [1, 2, 19].

Testing results were collated from two sources: (i) directly from veterinary practices testing patients in-clinic (SNAP® HW RT Test and SNAP® 4Dx® Plus Test) and (ii) through the IDEXX Reference Laboratories network (PetChek® Heartworm PF and Lab 4Dx® Plus Test). Results from veterinary practices were recorded in IDEXX VetLab® Instrumentation and Software and either automatically recorded by the IDEXX SnapShot Dx® Instrument or SNAP Pro® Analyzer or manually entered by clinic staff. To ensure data privacy, results were collected without owner information or canine patient identification and thus repeat testing events or translocated dogs (i.e. dogs with a travel history to another region) cannot be identified or omitted.

### Data analysis

State and county of the veterinary hospital providing the test result or submitting the sample to the laboratory were used to assign results to region as previously described [1, 2, 20], with four primary regional groups (Midwest, Northeast, Southeast, and West) used to compare results to previous publications. Percent positive results were calculated by dividing the number of tests reported as positive for each agent by the total number of testing events recorded in each county, state, or region. For state-wide summary tables and comparison to human disease reports, all results collected from 2013 to 2019 were included. For construction of maps depicting percent positive test results by county, individual counties with fewer than 210 total test results, or fewer than an average of 30 test results per study year, were excluded [1, 2] and then maps created with R (version 3.6.1) [21] using the albersusa [22] and tmap [23] packages.

### Statistical analyses

Reported total human cases in each state of Lyme borreliosis, ehrlichiosis, and anaplasmosis as documented in Centers for Disease Control and Prevention (CDC) Summary of Notifiable Infectious Diseases, 2009 through 2018, inclusive [24], were adjusted to reflect reported cases per 100,000 people using intercensal estimates of average state population data from the United States Census Bureau [25]; the 2019 CDC Summary of Notifiable Infectious Diseases was not available when analysis was performed. State-by-state comparison of population-adjusted human disease reports with canine seroprevalence was performed for each respective agent (*B. burgdorferi*, *Ehrlichia* spp., and *Anaplasma* spp.) using linear regression with significance assigned at *p* < 0.0001 and the coefficient of determination (*R*^2^) and *F* statistic calculated using Excel 2016 (Microsoft, Redmond, WA).

## Results

### Summary

**Fig. 1:**
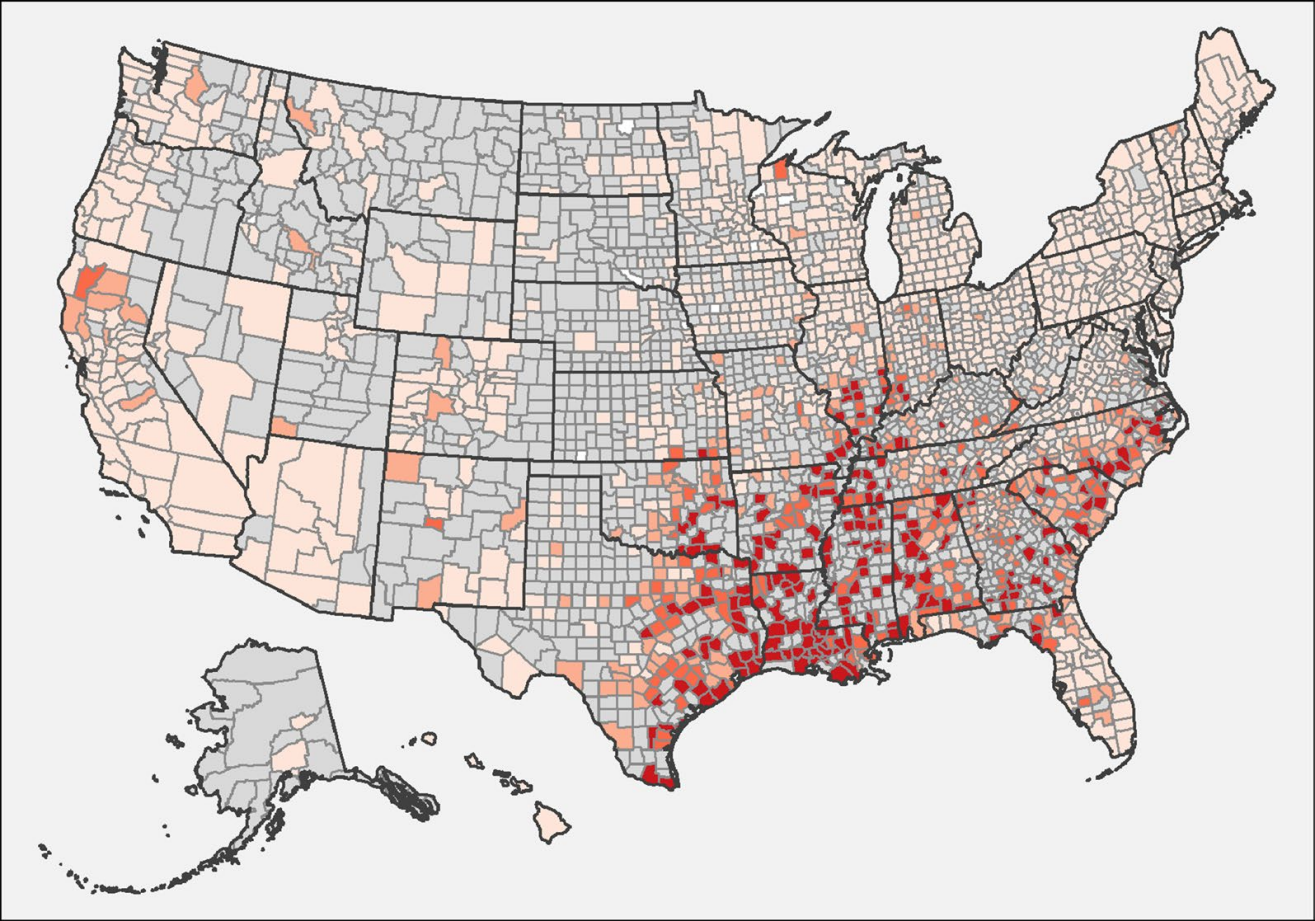
Evidence of antigen of *Dirofilaria immitis* in dogs by county, grouped according to percent positive tests. Few results (< 30 per year) were received from counties shaded gray, precluding interpretation of the presence of antigen in dogs from these areas. Counties with at least 210 results available for the 7-year period were shaded according to the following code: no dogs reported as positive (0%, white), 0.1–2.0% (light pink), 2.1–4.0% (pink), 4.1–6.0% (red), and > 6.0% (dark red)

**Fig. 2:**
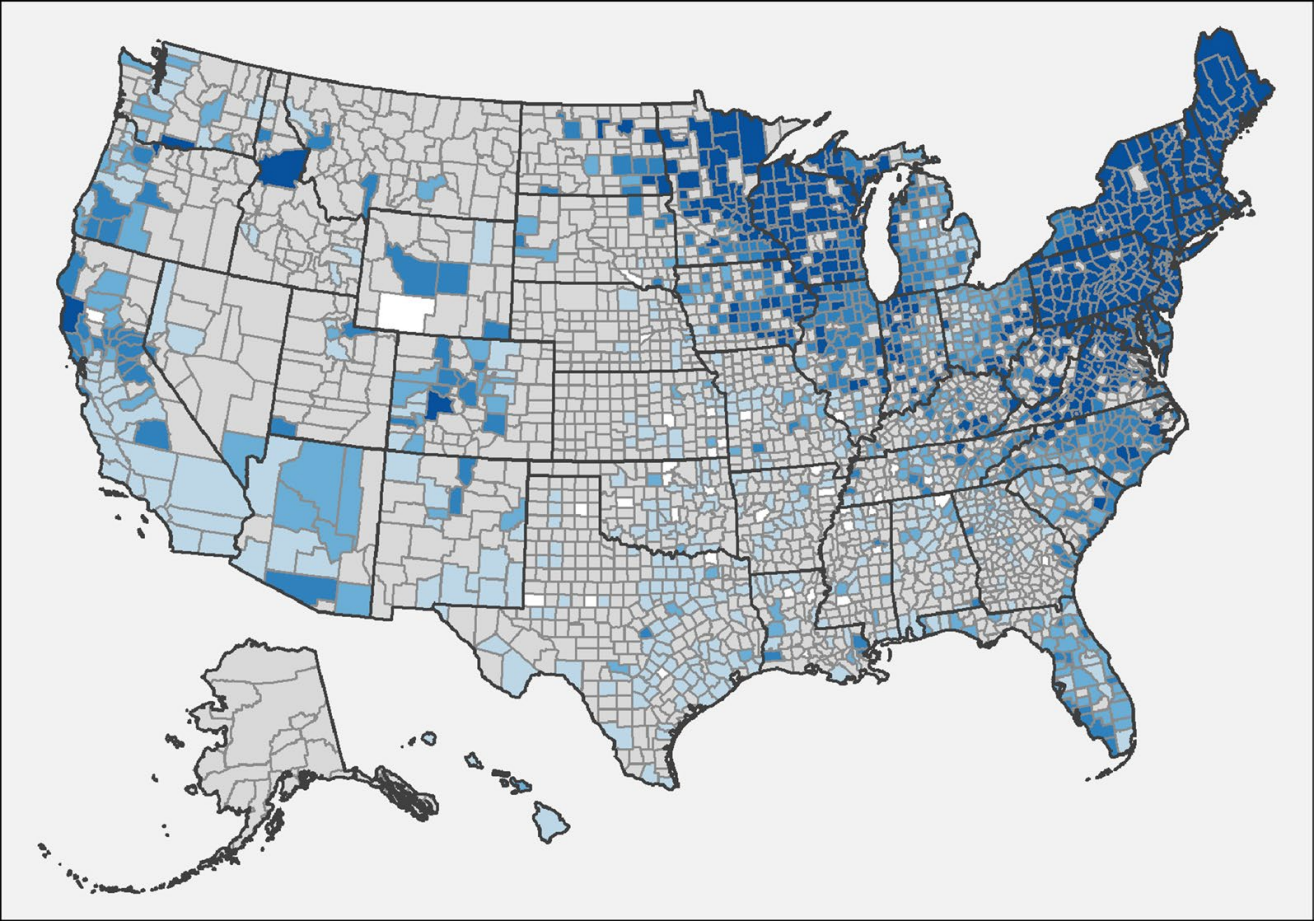
Evidence of antibody to *Borrelia burgdorferi* in dogs by county, grouped according to percent positive tests. Few results (< 30 per year) were received from counties shaded gray, precluding interpretation of the presence of antibody in dogs from these areas. Counties with at least 210 results available for the 7-year period were shaded according to the following code: no dogs reported as positive (0%, white), 0.1–0.5% (light blue), 0.5–1.0% (blue), 1.1–5.0% (dark blue), and > 5.0% (very dark blue)

**Fig. 3:**
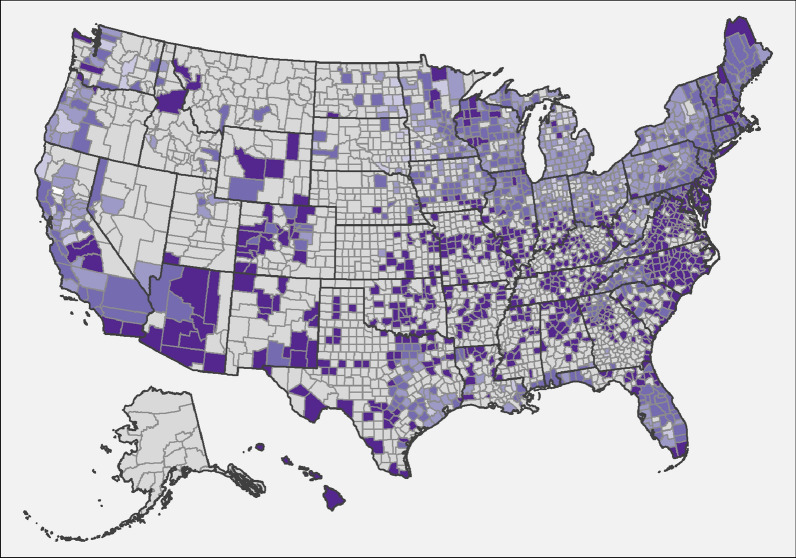
Evidence of antibody to *Ehrlichia* spp. in dogs by county, grouped according to percent positive tests. Few results (< 30 per year) were received from counties shaded gray, precluding interpretation of the presence of antibody in dogs from these areas. Counties with at least 210 results available for the 7-year period were shaded according to the following code: no dogs reported as positive (0%, white), 0.1–0.5% (light purple), 0.5–1.0% (purple), 1.1–2.0% (dark purple), and > 2.0% (very dark purple).

**Fig. 4.**
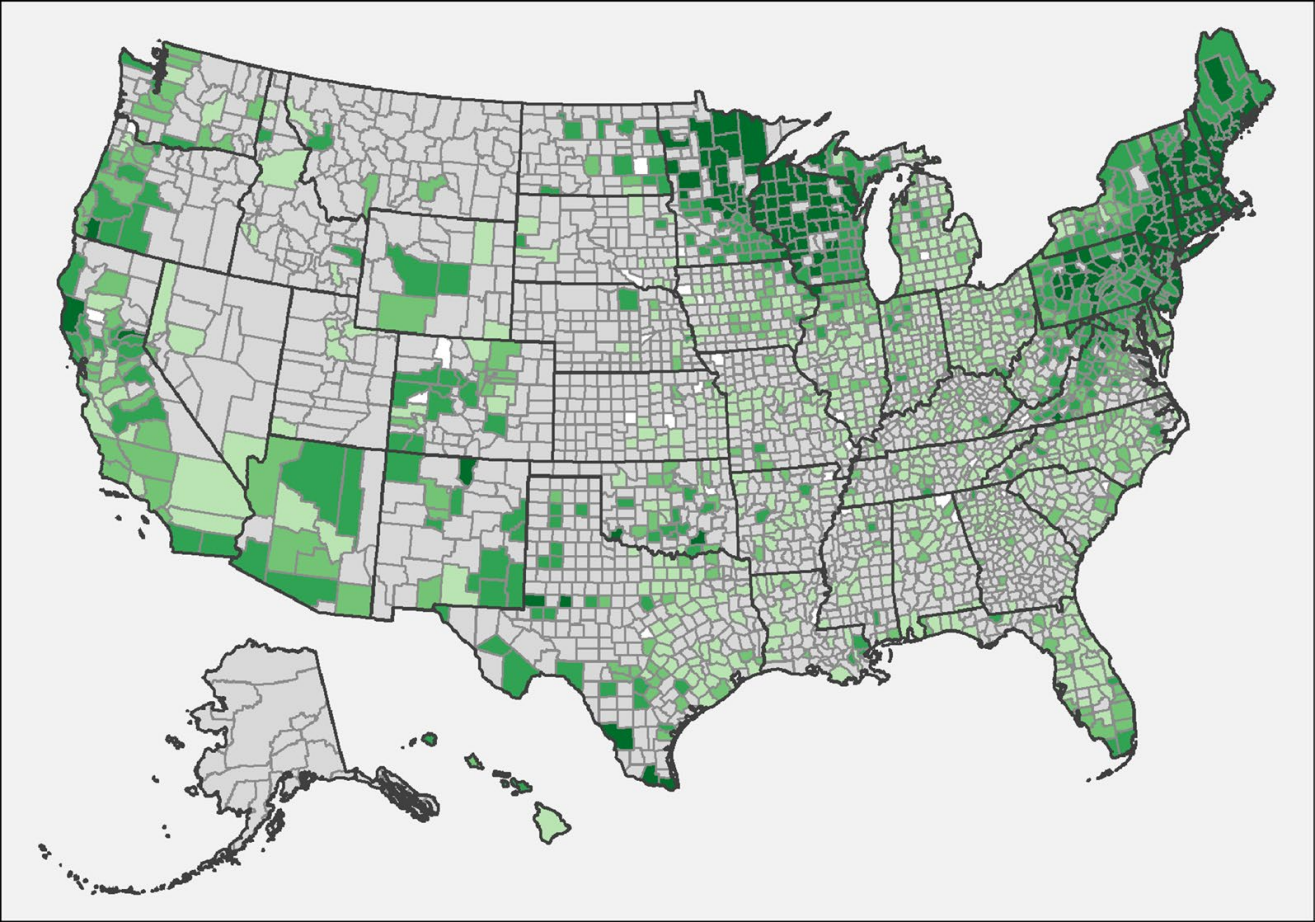
Evidence of antibody to *Anaplasma* spp. in dogs by county, grouped according to percent positive tests. Few results (< 30 per year) were received from counties shaded gray, precluding interpretation of the presence of antibody in dogs from these areas. Counties with at least 210 results available for the 7-year period were shaded according to the following code: no dogs reported as positive (0%, white), 0.1–0.5% (light green), 0.5–1.0% (green), 1.1–5.0% (dark green), and > 5.0% (very dark green)

A total of 144,022,232 results were available from dogs tested in 2298 counties and in all 50 states in the US over the 7-year period summarized in the present paper (Tables 1, 2, 3, 4). This represented results from nearly 50 million tests for antigen of *D. immitis* and more than 30 million tests for antibodies to *B. burgdorferi*, *Ehrlichia* spp., and *Anaplasma* spp. Evidence of all four agents was found in dogs from every state considered. Distribution of positive tests and relative percent positive values by county and state are shown in Figs. 1, 2, 3, 4.

**Table 1:** *Dirofilaria immitis* antigen percent positive test results (number positive/number tested) by region and state from dogs tested in the US from 2001 to 2007 [1], 2010 to 2012 [2], and 2013 to 2019

State	2001–2007 [1]	2010–2012 [2]	2013–2019
Northeast			
Connecticut	0.6% (236/37,650)	0.6% (1334/234,840)	0.6% (6016/1,043,798)
Delaware	1.6% (79/4986)	0.9% (449/51,497)	0.5% (905/191,348)
District of Columbia	NR	0.8% (54/7065)	0.7% (509/71,237)
Maine	0.6% (173/27,247)	0.3% (699/222,119)	0.5% (3235/639,514)
Maryland	0.8% (221.28,770)	0.4% (1159/286,989)	0.4% (5169/1,215,646)
Massachusetts	0.7% (1657/252,281)	0.5% (2570/512,302)	0.6% (10,680/1,863,965)
New Hampshire	0.8% (168/21,056)	0.4% (556/131,724)	0.6% (3268/559,139)
New Jersey	0.3% (384/111,245)	0.3% (1094/356,617)	0.4% (5056/1,205,338)
New York	0.5% (780/158,926)	0.4% (2281/620,933)	0.4% (10,486/2,564,677)
Pennsylvania	0.4% (191/45,815)	0.2% (946/623,058)	0.4% (8818/2,334,505)
Rhode Island	0.8% (123/16,199)	0.4% (274/67,811)	0.7% (1097/158,349)
Vermont	0.7% (24/3682)	0.4% (259/60,125)	0.7% (1429/217,697)
Region	0.6% (4036/707,857)	0.4% (11,675/3,175,080)	0.5% (56,668/12,065,213)
Midwest			
Illinois	0.9% (2915/337,434)	0.7% (4367/614,303)	1.0% (23,338/2,414,323)
Indiana	1.8% (428/24,290)	1.4% (2834/207,125)	1.3% (13,158/980,336)
Iowa	0.9% (164/19,097)	0.3% (401/125,196)	0.4% (2466/559,015)
Kansas	2.7% (170/6264)	1.0% (845/80,174)	0.9% (3150/361,642)
Michigan	0.7% (2031/292,171)	0.8% (4129/490,541)	0.7% (17,232/2,361,699)
Minnesota	0.4% (332/80,810)	0.3% (693/279,699)	0.4% (4406/1,097,589)
Missouri	2.0% (457/22,673)	1.5% (2615/173,842)	1.6% (14,505/898,730)
Nebraska	0.8% (34/4387)	0.7% (126/17,613)	0.5% (801/149,756)
North Dakota	0.5% (25/4914)	0.1% (16/17,100)	0.3% (303/99,478)
Ohio	0.9% (1242/136,548)	0.6% (2717/448,847)	0.6% (11,706/2,107,379)
South Dakota	0.1% (1/962)	0.3% (45/13,831)	0.4% (277/79,978)
Wisconsin	0.6% (616/109,745)	0.4% (1226/349,644)	0.4% (5940/1,331,056)
Region	0.8% (8415/1,039,295)	0.7% (20,014/2,817,915)	0.8% (97,282/12,440,981)
Southeast			
Alabama	3.4% (622/18,388)	3.6% (4479/125,156)	3.9% (24,240/629,814)
Arkansas	6.8% (578/8526)	4.6% (3402/74,386)	4.0% (17,793/442,206)
Florida	1.8% (1408/80,280)	1.6% (11,189/696,358)	1.4% (41,922/3,058,920)
Georgia	2.7% (1373/51,494)	3.2% (10,142/317,138)	2.6% (39,669/1,550,488)
Kentucky	1.1% (227/20,092)	1.8% (1383/75,835)	1.4% (7069/518,778)
Louisiana	6.0% (871/14,468)	7.4% (7133/96,223)	7.2% (46,444/641,766)
Mississippi	7.4% (183/2459)	10.5% (2835/26,988)	8.1% (21,499/265,613)
North Carolina	3.0% (663/22,005)	2.4% (8772/373,078)	2.2% (50,390/2,326,822)
Oklahoma	2.1% (254/11,913)	3.4% (2657/79,056)	2.2% (9737/439,331)
South Carolina	5.7% (860/15,019)	2.9% (4640/160,252)	2.6% (23,949/906,389)
Tennessee	3.6% (498/13,787)	3.4% (6937/204,231)	2.6% (23,956/936,084)
Texas	5.5% (12,160/220,829)	3.9% (34,066/872,096)	3.5% (140,198/3,990,180)
Virginia	1.1% (331/29,766)	1.0% (3827/384,074)	0.9% (17,702/2,038,858)
West Virginia	0.8% (51/6131)	0.5% (388/77,319)	0.4% (1205/274,360)
Region	3.9% (20,079/515,157)	2.9% (101,850/3,562,190)	2.6% (465,773/18,019,609)
West			
Alaska	NR	2.3% (13/566)	1.4% (54/3,768)
Arizona	1.2% (620/53,809)	0.6% (1005/156,152)	0.5% (3577/657,405)
California	1.6% (8478/530,788)	0.8% (4850/649,681)	0.6% (15,265/2,446,653)
Colorado	0.4% (1028/261,358)	0.7% (1182/171,057)	0.9% (5961/690,515)
Hawaii	NR	1.5% (240/16,548)	0.7% (816/124,893)
Idaho	0.6% (32/5748)	0.5% (68/14,253)	0.4% (210/48,509)
Montana	0.6% (16/2801)	0.5% (23/4950)	0.7% (165/23,827)
Nevada	1.2% (74/6180)	0.5% (165/32,951)	0.6% (688/119,315)
New Mexico	1.8% (427/23,429)	1.5% (761/49,628)	1.4% (2077/149,284)
Oregon	0.8% (235/29,176)	0.7% (344/51,964)	0.6% (1355/237,911)
Utah	0.6% (11/1,904)	0.7% (73/10,886)	0.5% (248/50,016)
Washington	1.0% (39/4,099)	0.9% (136/16,026)	0.6% (986/157,038)
Wyoming	1.2% (10/700)	0.6% (27/4285)	1.1% (222/19,882)
Region	1.2% (10,970/919,992)	0.8% (8887/1,178,947)	0.7% (31,624/4,729,016)
Overall	1.4% (43,500/3,182,301)	1.3% (142,426/10,734,132)	1.4% (651,347/47,254,819)

**Table 2:** *Borrelia burgdorferi* antibody percent positive test results (number positive/number tested) by region and state from dogs tested in the US from 2001 to 2007 [1], 2010 to 2012 [2], and 2013 to 2019

State	2001–2007 [1]	2010–2012 [2]	2013–2019
Northeast			
Connecticut	18.1% (1846/10,209)	18.0% (33,071/183,787)	15.5% (134,875/871,389)
Delaware	11.2% (516/4595)	9.5% (4671/49,126)	5.9% (10,934/186,675)
District of Columbia	NR	8.2% (574/7029)	8.9% (6227/69,650)
Maine	11.6% (3269/28,230)	13.5% (29,860/221,556)	13.8% (87,442/635,002)
Maryland	12.6% (2882/22,945)	10.0% (27,348/273,406)	6.9% (80,393/1,157,374)
Massachusetts	19.8% (6729/33,915)	18.3% (74,429/406,493)	15.3% (256,688/1,679,429)
New Hampshire	12.9% (2343/18,122)	15.8% (20,447/129,842)	13.1% (73,199/556,671)
New Jersey	14.2% (2913/20,575)	13.1% (38,695/295,084)	9.8% (102,967/1,047,889)
New York	7.1% (5781/81,305)	9.5% (50,802/536,978)	10.5% (241,549/2,305,462)
Pennsylvania	9.4% (3869/40,948)	12.9% (74,481/579,657)	13.2% (291,604/2,211,655)
Rhode Island	14.3% (933/6,508)	15.7% (10,001/63,797)	12.9% (18,672/144,716)
Vermont	9.9% (368/3,718)	14.8% (8833/59,518)	14.3% (30,759/215,341)
Region	11.6% (31,449/271,070)	13.3% (373,212/2,806,273)	12.1% (1,335,309/11,081,253)
Midwest			
Illinois	1.0% (324/31,976)	3.0% (8413/277,352)	2.8% (44,155/1,554,905)
Indiana	1.1% (231/20,515)	3.5% (3961/112,480)	3.7% (26,769/721,305)
Iowa	0.9% (149/17,390)	2.9% (3236/111,522)	3.8% (17,822/472,821)
Kansas	0.1% (6/5473)	0.5% (263/52,435)	0.2% (458/217,196)
Michigan	0.6% (431/67,625)	1.2% (2936/236,875)	1.5% (24,299/1,569,693)
Minnesota	9.5% (7267/76,610)	8.6% (20,159/234,564)	7.9% (75,183/955,737)
Missouri	0.2% (59/24,095)	0.6% (616/108,580)	0.4% (1722/477,732)
Nebraska	0.1% (5/4282)	2.0% (91/4489)	0.4% (222/51,900)
North Dakota	3.0% (136/4558)	5.4% (893/16,560)	4.4% (3,898/88,388)
Ohio	0.2% (140/61,138)	0.7% (1970/278,493)	1.4% (21,577/1,513,499)
South Dakota	0.3% (1/358)	6.0% (270/4,497)	0.7% (376/53,478)
Wisconsin	10.2% (6018/59,070)	11.8% (33,217/282,663)	8.7% (100,656/1,162,272)
Region	4.0% (14,767/373,090)	4.4% (76,025/1,720,510)	3.6% (317,137/8,838,926)
Southeast			
Alabama	0.1% (27/18,998)	0.7% (367/53,340)	0.3% (879/284,182)
Arkansas	0.1% (7/8391)	0.5% (220/42,776)	0.3% (420/159,161)
Florida	0.5% (256/54,982)	1.0% (3832/403,886)	0.7% (11,285/1,588,284)
Georgia	0.3% (77/23,333)	0.8% (985/124,665)	0.4% (2808/707,236)
Kentucky	0.2% (45/18,935)	1.5% (847/56,049)	1.5% (5367/346,951)
Louisiana	0.1% (9/11,197)	0.4% (48/12,449)	0.4% (456/120,604)
Mississippi	0.0% (1/2198)	0.7% (43/6643)	0.2% (152/68,995)
North Carolina	1.3% (263/20,783)	1.9% (4,837/249,170)	2.2% (40,435/1,797,246)
Oklahoma	0.2% (19/11,549)	0.6% (445/70,753)	0.2% (621/301,050)
South Carolina	1.3% (148/11,562)	1.0% (857/82,684)	1.2% (5296/453,004)
Tennessee	0.2% (47/18,891)	0.6% (670/111,314)	0.7% (3247/456,799)
Texas	0.2% (91/58,088)	0.5% (1935/432,919)	0.2% (4038/1,778,128)
Virginia	6.7% (1924/28,787)	9.7% (33,994/350,489)	7.9% (148,215/1,886,576)
West Virginia	0.3% (9/2942)	3.5% (2152/61,437)	8.0% (20,034/249,841)
Region	1.0% (2923/290,636)	2.5% (51,232/2,058,574)	2.4% (243,253/10,198,057)
West			
Alaska	NR	NR	3.6% (16/444)
Arizona	0.4% (4/992)	0.8% (424/55,893)	0.7% (2,421/345,481)
California	1.8% (540/29,454)	1.6% (4447/270,516)	1.0% (13,425/1,280,960)
Colorado	0.4% (49/11,557)	1.0% (192/19,489)	0.8% (1354/175,609)
Hawaii	NR	0.3% (6/2360)	0.2% (122/51,379)
Idaho	0.3% (1/369)	3.6% (6/169)	0.7% (107/15,917)
Montana	NR	0 (0/37)	1.4% (79/5842)
Nevada	NR	0.6% (74/12,286)	0.6% (178/31,574)
New Mexico	0.3% (7/2060)	0.7% (185/26,714)	0.4% (316/72,549)
Oregon	2.8% (77/2798)	1.7% (312/17,893)	1.0% (1110/115,018)
Utah	0 (0/93)	1.2% (9/784)	0.8% (62/8231)
Washington	0 (0/33)	1.5% (64/4338)	0.5% (314/60,704)
Wyoming	0 (0/184)	1.9% (7/361)	0.9% (47/4968)
Region	1.4% (678/47,540)	1.4% (5726/410,840)	0.9% (19,551/2,168,676)
Overall	5.1% (49,817/982,336)	7.2% (509,195/6,996,197)	5.9% (1,915,250/32,286,912)

**Table 3:** *Ehrlichia* spp. antibody percent positive test results (number positive/number tested) by region and state from dogs tested in the US from 2001 to 2007 [1], 2010 to 2012 [2], and 2013 to 2019

State	2001–2007 [1]	2010–2012 [2]	2013–2019
Northeast			
Connecticut	0.2% (21/10,209)	0.8% (1434/183,776)	1.5% (13,159/870,092)
Delaware	1.0% (48/4595)	2.3% (1,114/49,131)	4.9% (9075/186,675)
District of Columbia	NR	1.6% (113/7029)	3.8% (2679/69,650)
Maine	0.1% (39/28,230)	0.6% (1214/221,555)	1.4% (8909/635,005)
Maryland	0.7% (165/22,945)	1.9% (5107/273,382)	4.6% (52,771/1,157,266)
Massachusetts	0.3% (107/33,915)	0.8% (3315/406,476)	1.8% (30,583/1,679,373)
New Hampshire	0.2% (36/18,122)	0.7% (949/129,829)	1.7% (9499/556,670)
New Jersey	0.4% (89/20,575)	1.2% (3638/295,047)	3.1% (32,278/1,046,276)
New York	0.2% (179/81,305)	0.6% (3176/536,968)	1.4% (32,835/2,304,678)
Pennsylvania	0.2% (80/40,948)	0.6% (3364/579,608)	1.1% (24,835/2,211,622)
Rhode Island	0.1% (6/6508)	0.3% (206/63,796)	1.4% (1958/144,718)
Vermont	0.2% (7/3718)	0.6% (381/59,515)	1.6% (3420/215,341)
Region	0.3% (777/271,070)	0.9% (24,011/2,806,112)	2.0% (222,001/11,077,366)
Midwest			
Illinois	0.4% (135/31,976)	0.8% (2155/277,174)	1.8% (28,173/1,554,356)
Indiana	0.3% (54/20,515)	1.3% (1480/112,477)	1.8% (13,048/721,278)
Iowa	0.4% (61/17,390)	0.7% (751/111,518)	1.4% (6435/472,492)
Kansas	2.2% (119/5,473)	2.3% (1228/52,429)	4.6% (9982/217,197)
Michigan	0.1% (34/67,625)	0.3% (781/236,798)	0.6% (10,126/1,569,688)
Minnesota	0.3% (202/76,610)	0.6% (1426/234,558)	1.2% (11,517/955,740)
Missouri	1.9% (462/24,095)	5.4% (5888/108,573)	10.4% (49,485/477,734)
Nebraska	0.3% (13/4282)	1.6% (70/4485)	1.8% (956/51,900)
North Dakota	0.0% (1/4558)	0.3% (55/16,560)	0.6% (496/88,388)
Ohio	0.1% (79/61,138)	0.6% (1727/278,437)	1.1% (16,838/1,513,496)
South Dakota	0 (0/358)	0.6% (25/4497)	0.5% (286/53,477)
Wisconsin	0.3% (194/59,070)	0.6% (1751/282,662)	1.2% (13,900/1,162,273)
Region	0.4% (1354/373,090)	1.0% (17,337/1,720,168)	1.8% (161,242/8,838,019)
Southeast			
Alabama	0.3% (64/18,998)	1.6% (856/53,339)	3.5% (10,024/284,181)
Arkansas	3.9% (324/8391)	9.4% (4029/42,774)	18.4% (29,341/159,161)
Florida	0.8% (425/54,982)	1.2% (4644/403,622)	1.8% (28,518/1,588,286)
Georgia	1.9% (444/23,333)	2.6% (3290/124,637)	3.7% (26,377/707,157)
Kentucky	0.8% (152/18,935)	4.3% (2420/56,027)	6.9% (23,837/346,952)
Louisiana	0.2% (18/11,197)	1.1% (140/12,406)	1.9% (2264/120,602)
Mississippi	3.1% (68/2198)	4.6% (308/6637)	7.0% (4841/68,995)
North Carolina	2.1% (431/20,783)	4.6% (11,431/249,132)	6.4% (115,377/1,797,218)
Oklahoma	3.8% (439/11,549)	5.4% (3,847/70,751)	8.3% (24,845/301,051)
South Carolina	0.8% (95/11,562)	1.4% (1151/82,677)	2.6% (11,655/453,006)
Tennessee	2.3% (428/18,891)	3.0% (3307/111,312)	8.1% (37,150/456,799)
Texas	0.8% (441/58,088)	1.8% (7659/432,799)	2.2% (38,424/1,778,110)
Virginia	1.8% (532/28,787)	6.2% (21,770/350,437)	9.4% (176,931/1,886,580)
West Virginia	0.1% (4/2942)	0.6% (339/61,434)	1.2% (3091/249,842)
Region	1.3% (3865/290,636)	3.2% (65,191/2.057,984)	5.2% (532,675/10,197,940)
West			
Alaska	NR	NR	3.8% (17/444)
Arizona	3.2% (32/992)	2.4% (1349/55,865)	2.8% (9628/34,5481)
California	0.8% (225/29,454)	0.8% (2258/270,190)	1.1% (14,600/1,280,960)
Colorado	0.2% (19/11,557)	1.1% (217/19,467)	2.2% (3842/175,609)
Hawaii	NR	7.0% (166/2359)	2.7% (1368/51,378)
Idaho	0 (0/369)	0.6% (1/167)	1.0% (167/15,917)
Montana	NR	0 (0/36)	1.5% (86/5842)
Nevada	NR	0.5% (59/12,278)	0.9% (284/31,574)
New Mexico	1.0% (21/2060)	3.2% (858/26,706)	3.3% (2393/72,549)
Oregon	0.1% (2/2798)	0.6% (111/17,879)	0.9% (1043/115,016)
Utah	0 (0/93)	0.5% (4/783)	1.0% (79/8231)
Washington	0 (0/33)	2.5% (109/4330)	1.2% (734/60,702)
Wyoming	0 (0/184)	0.6% (2/359)	2.7% (136/4968)
Region	0.6% (299/47,540)	1.3% (5134/410,419)	1.6% (34,377/2,168,671)
Overall	0.6% (6295/982,336)	1.6% (111,673/6,994,683)	2.9% (950,295/32,281,996)

**Table 4:** *Anaplasma* spp. antibody percent positive test results (number positive/number tested) by region and state from dogs tested in the US from 2001 to 2007 [1], 2010 to 2012 [2], and 2013 to 2019

State	2001–2007 [1]	2010–2012 [2]	2013–2019
Northeast			
Connecticut	21.8% (1499/6887)	20.3% (37,390/183,792)	15.4% (134,039/870,093)
Delaware	1.1% (48/4315)	1.0% (460/47,722)	0.9% (1704/183,604)
District of Columbia	NR	1.6% (57/3687)	1.6% (1096/69,650)
Maine	5.4% (1341/24,632)	8.3% (18,367/220,977)	11.4% (72,231/634,959)
Maryland	1.7% (282/16,307)	1.6% (3887/239,461)	1.3% (15,227/1,149,654)
Massachusetts	10.4% (2803/26,911)	10.7% (41,223/385,659)	12.4% (208,757/1,678,729)
New Hampshire	4.5% (618/13,743)	7.7% (9605/125,054)	11.1% (61,104/556,329)
New Jersey	9.8% (1339/13,721)	8.5% (24,330/286,133)	6.3% (65,488/1,046,199)
New York	3.6% (1741/48,201)	6.1% (30,916/506,075)	5.9% (135,113/2,304,237)
Pennsylvania	1.6% (449/27,641)	2.5% (13,585/536,513)	3.7% (81,660/2,209,906)
Rhode Island	4.7% (158/3396)	12.8% (7477/58,211)	9.9% (14,368/144,485)
Vermont	1.7% (46/2684)	3.7% (2189/59,517)	7.9% (17,096/215,341)
Region	5.5% (10,324/188,438)	7.1% (189,486/2,652,801)	7.3% (807,883/11,063,186)
Midwest			
Illinois	0.4% (51/11,899)	1.0% (2369/249,268)	0.6% (9947/1,553,848)
Indiana	0.4% (26/7084)	0.5% (436/86,974)	0.4% (2755/720,622)
Iowa	0.4% (21/4840)	0.7% (742/110,278)	0.5% (2451/472,000)
Kansas	0.5% (7/1452)	0.4% (191/49,142)	0.2% (492/217,126)
Michigan	1.2% (190/16,312)	0.5% (1149/214,347)	0.5% (8458/1,569,034)
Minnesota	9.8% (6002/61,374)	9.5% (22,338/234,565)	6.0% (57,553/955,732)
Missouri	0.3% (14/5250)	0.4% (263/73,963)	0.3% (1498/466,532)
Nebraska	NR	0.9% (36/4143)	0.6% (289/51,759)
North Dakota	2.4% (40/1692)	3.4% (557/16,556)	2.6% (2265/88,388)
Ohio	0.1% (13/14,414)	0.3% (691/223,187)	0.3% (4700/1,507,641)
South Dakota	NR	12.7% (456/3599)	0.4% (193/50,497)
Wisconsin	10.5% (5,409/51,512)	10.7% (30,352/282,664)	6.1% (71,335/1,162,247)
Region	6.7% (11,773/175,829)	3.9% (59,580/1,548,686)	1.8% (161,936/8,815,426)
Southeast			
Alabama	0.1% (4/4331)	0.3% (150/46,258)	0.3% (703/276,999)
Arkansas	0.6% (10/1743)	0.5% (178/37,465)	0.3% (531/158,769)
Florida	0.5% (166/31,690)	0.7% (2375/327,715)	0.6% (9277/1,581,448)
Georgia	0.2% (15/8856)	0.4% (297/81,133)	0.2% (1747/705,485)
Kentucky	0.1% (5/4319)	0.5% (165/31,795)	0.3% (1052/339,065)
Louisiana	0.1% (1/707)	0.3% (28/9501)	0.4% (493/120,074)
Mississippi	0 (0/300)	0.6% (33/5295)	0.3% (176/68,942)
North Carolina	0.4% (25/6841)	0.6% (1076/196,723)	0.4% (7947/1,794,296)
Oklahoma	1.2% (70/5920)	1.0% (650/68,486)	0.6% (1858/300,920)
South Carolina	0.1% (9/6507)	0.5% (188/41,085)	0.4% (1834/451,566)
Tennessee	0.1% (4/4324)	0.3% (284/82,326)	0.3% (1387/456,109)
Texas	0.6% (90/14,788)	1.2% (3888/336,473)	0.9% (15,137/1,771,495)
Virginia	0.9% (96/10,195)	1.4% (4402/311,594)	1.0% (18,448/1,883,167)
West Virginia	0.2% (1/627)	0.6% (332/55,483)	0.9% (2140/248,376)
Region	0.5% (496/101,148)	0.9% (14,046/1,631,332)	0.6% (62,730/10,156,711)
West			
Alaska	NR	NR	0.9% (4/444)
Arizona	0.7% (4/583)	0.6% (259/40,490)	0.8% (2669/343,274)
California	4.8% (612/12,673)	2.3% (5,571/255,781)	1.4% (17,779/1,279,877)
Colorado	0 (0/86)	1.1% (120/11,145)	0.7% (1,271/174,234)
Hawaii	NR	0.9% (18/1920)	0.6% (309/51,265)
Idaho	0.7% (2/298)	1.2% (2/169)	0.43 (69/15,917)
Montana	NR	5.4% (2/37)	0.9% (55/5841)
Nevada	NR	0.4% (31/8456)	0.3% (97/31,243)
New Mexico	0.3% (1/289)	1.7% (341/20,344)	1.2% (850/72,324)
Oregon	7.4% (22/296)	3.0% (475/15,807)	1.6% (1857/1,149,46)
Utah	NR	0.8% (6/784)	0.4% (35/8222)
Washington	NR	1.2% (49/4162)	0.7% (444/60,629)
Wyoming	NR	0.6% (2/354)	0.8% (38/4,966)
Region	4.5% (641/14,225)	2.0% (7056/359,449)	1.2% (25,477/2,163,182)
Overall	4.8% (23,234/479,640)	4.4% (270,168/6,192,268)	3.3% (1,058,026/32,198,505)

### Heartworm

Percent positive test results for *D. immitis* antigen were higher in the Southeast than in the other three regions and were higher in the West and Midwest than in the Northeast (Table 1, Fig. 1). National and regional prevalence of percent positive test results for *D. immitis* was largely unchanged from our previous reports [1, 2] with the exception of the Southeast, where the overall prevalence was lower than in previous reports; this decrease in prevalence was evident in every state in the Southeast except Alabama (Table 1).

### Lyme disease

Percent positive test results for antibody to *B. burgdorferi* were highest in the Northeast, followed by the Midwest, Southeast, and West (Table 2, Fig. 2). The overall, national prevalence of antibodies to *B. burgdorferi* was decreased from our previous report (from 7.2 to 5.9%) and was lower in every region except the Southeast where percent positive test results remained unchanged (Table 2). Prevalence of antibodies to *B. burgdorferi* for most individual states remained unchanged or decreased; only New York, Iowa, Ohio, and West Virginia were significantly higher than the earlier report (Table 2).

### Ehrlichiosis

Percent positive test results for antibody to *Ehrlichia* spp. were higher in the Southeast than in the other three regions (Table 3, Fig. 3). National and regional canine seroprevalence for *Ehrlichia* spp. antibodies was also higher than in previous reports, including twofold higher in the Northeast and as much as threefold higher in the Southeast (Table 3). Prevalence of *Ehrlichia* spp. antibody was also higher in every state in the Northeast and Southeast, every state in the Midwest except South Dakota, and all states in West except Hawaii, New Mexico, and Washington (Table 3).

### Anaplasmosis

Percent positive test results for antibody to *Anaplasma* spp. were highest in the Northeast and lowest in the Southeast (Table 4). Overall, seroprevalence of antibodies to *Anaplasma* spp. decreased nationally (from 4.4 to 3.3%) and in every region except the Northeast, where it remained largely unchanged from the previous report (Table 4). By state, percent positive test results for *Anaplasma* spp. antibodies were lower in states throughout the Southeast, Midwest, and West. In the Northeast, statewide seroprevalence was higher in northern New England (Maine, New Hampshire, Vermont) but lower in several mid-Atlantic states (Connecticut, New Jersey, Rhode Island) (Table 4).

### Comparison to human disease reports

Seroprevalence of *B. burgdorferi* antibodies in dogs and reported cases of Lyme borreliosis in people by state were positively associated (*R*^2^ = 0.812, *F* = 207.0). Reported human cases of Lyme borreliosis were lower than expected based on canine seroprevalence in Massachusetts, New York, Virginia, and West Virginia, and higher than expected in Delaware, Maine, New Hampshire, and Vermont. Seroprevalence of *Ehrlichia* spp. antibodies in dogs and reported cases of ehrlichiosis in people by state were positively associated (*R*^2^ = 0.521, *F* = 52.2). In some states, reported human cases of ehrlichiosis were lower than expected (Mississippi) based on canine serology. Seroprevalence of *Anaplasma* spp. antibodies in dogs and reported cases of anaplasmosis in people by state were positively associated (*R*^2^ = 0.546, *F* = 57.6). In some states, reported human cases of anaplasmosis were lower (Connecticut and Massachusetts) or higher (Vermont) than expected based on canine serology.

## Discussion

The present study is the third in a series which taken together describe nearly 2 decades of results and constitutes the largest ever reported survey of dogs for multiple vector-borne infections, expanding on our previous publications by including data from the past 7 years and 4- to 30-times more canine test results [1, 2]. These data represent findings from annual testing of approximately 7% to 12% of the estimated 60 million pet dogs that receive annual veterinary care in the US [26] and thus provide valuable insights into canine infection with heartworm, infestation with ticks, and past or current infection with several tick-borne disease agents. Positive results for all four canine vector-borne agents considered (heartworm, *Borrelia burgdorferi*, *Ehrlichia* spp., and *Anaplasma* spp.) were found in every state in the US (Tables 1, 2, 3, 4), the first time we have observed national evidence of all four types of infections in the 17 years of data included in these surveys [1, 2]. Because data are anonymized, we cannot exclude the possibility of repeated testing events or of identifying evidence of infections acquired in a different geographic location. Nonetheless, a number of key trends are evident in the geographic distribution (Figs. 1, 2, 3, 4) and relative prevalence of percent positive results in different regions (Tables 1, 2, 3, 4).

For canine heartworm infection as determined by detection of antigen, the data in the present paper suggest that overall prevalence in well-cared-for pet dogs has remained largely unchanged since national surveys using similar sample sets were first reported [1] and that percent positive test results from pet dogs have decreased in southern states (Table 1), from 3.9% of test results from 2001 to 2007 [1] to 2.6% of test results from 2013 to 2019. Interestingly, during a similar time period, other researchers reported a significant increase in the prevalence of heartworm infection in the USA, the most marked of which was observed in the South [11, 12, 27, 28], although regional areas where prevalence was clearly decreasing were also identified [27]. Apparent discrepancies between the data in the present article and those reported by others may be due to differences in testing platforms, survey methods, or patient profile. The reports showing an apparent increase in percent positive test results included data from multiple diagnostic laboratories [29] whereas the present paper used only data from practices using well-validated IDEXX tests, which may be a source of selection bias. Surveys of the number of dogs treated for heartworm are useful but cannot be directly compared to infection prevalence trends, and artificial increases in percent positive results can be seen when testing is used primarily for diagnostic verification of a suspected infection rather than for routine screening [28, 29]. For example, the similar percent positive test results in dogs in Alaska (1.4%) and Florida (1.4%) in the present study likely reflect targeted testing by Alaska veterinarians based on a travel history compared to routine screening of dogs protected from infection by preventives despite intense transmission pressure in Florida [30]. Nonetheless, heartworm is a preventable infection, and the finding that 1.4% of pet dogs receiving veterinary care in the USA, or approximately 840,000 pet dogs overall, are identified as infected with this potentially fatal parasite each year, is dispiriting.

Canine infection with *B. burgdorferi* remains widespread, with antibodies detected in 5.1% of dogs in the present study overall and 12.1% of dogs in the Northeast. Significant increases in percent positive test results were evident in some areas of the upper South, including West Virginia and North Carolina, consistent with other reports of geographic spread of the maintenance cycle for this pathogen [10, 13, 31]. At the same time, decreased statewide seroprevalence of antibodies to *B. burgdorferi,* in some cases by more than 40%, was evident in several states where Lyme disease has long been endemic or hyperendemic, including Connecticut, Massachusetts, New Jersey, Delaware, and Maryland (Table 2), a trend that has been previously reported [10]. Canine infection with *B. burgdorferi* varies widely even in relatively focal areas [17] and can be prevented with a combination of tick control and vaccination [32]. Increased adherence to these recommendations, including the widespread use of systemic isoxazoline acaricides, which first became available in the USA in 2014, would be expected to result in decreased percent positive test results in dogs over time. However, *B. burgdorferi* infection remains common, particularly in areas of the Northeast where *I. scapularis* vector populations are intense or expanding, and canine seroprevalence to *B. burgdorferi* continues to increase in northern New England, western New York, and western Pennsylvania [10, 33]. In other regions, such as in states along the southern border of the USA, autochthonous transmission of *B. burgdorferi* has not been documented, but antibodies to *B. burgdorferi* may occasionally be identified in dogs that move to that region, creating confusion when survey results are not carefully interpreted in context [34, 35].

Results of the current serosurvey demonstrate an increase in the number of dogs with antibodies to *Ehrlichia* spp. in nearly all states within the US. Since the publication of the 2010–2012 seroprevalence results, the ELISA test for antibodies to *Ehrlichia* spp. was modified to include a new peptide for the detection of antibodies to *E. ewingii* [19]. Canine seroreactivity to this new target, as well as the increase in seropositive results observed particularly across the Southeast, is consistent with regional results from a study where canine serum samples were obtained from academic and private veterinary hospitals [6]. In that earlier study, antibodies to *E. canis* were more frequently detected in dogs in the southcentral region where the brown dog tick vectors predominate, while antibodies to both *E. ewingii* and *E. chaffeensis* were more prevalent in dogs from a band of states across the upper south, where *A. americanum* is most common [6]. Indeed, *A. americanum*, the tick responsible for transmitting *E. ewingii*, is highly prevalent across the Southeast region with evidence of geographic spread to states within the Midwest and Northeast [15, 36]. Even before the test could detect antibodies to *E. ewingii*, the Midwest region had evidence of canine seroreactivity to *Ehrlichia* spp. [1, 2]. It is not known if this previous and continued seroreactivity is associated with the novel *Ehrlichia muris* subsp. *euclairensis* reported there [37] since only one clinical infection with this pathogen has been documented in the dog [38]. Because the different *Ehrlichia* spp. antibodies are detected by reactivity on a single spot or microtiter well of the test, it is not possible to determine the infecting species of *Ehrlichia*. While geographic location and predominant tick species may provide some guidance, elucidating the causative *Ehrlichia* species is becoming increasingly difficult because of expanding tick ranges and the transport of dogs across the country for adoption.

The seroprevalence of *Anaplasma* spp., although decreased slightly in the Midwest and certain Northeastern states, remained largely unchanged relative to the previous report [2]. In much of the northern USA, antibodies to *Anaplasma* spp. (presumably to *A. phagocytophilum*) were detected less frequently than antibodies to *B. burgdorferi* in most states despite transmission by the same species of tick, *Ixodes scapularis*. Surveys conducted in areas of the Northeast and upper Midwest have found that 3–10 times as many *Ixodes scapularis* nymphs and adult ticks contain *B. burgdorferi* DNA compared to *A. phagocytophilum* DNA [39–41]. In a recent study which tested *I. scapularis* removed from dogs and cats in the Northeastern USA, 17.8% were infected with *B. burgdorferi* and 2.6% with *A. phagocytophilum* [42]. A greater proportion of ticks harboring *B. burgdorferi* could make it more likely that dogs would be exposed to this pathogen and perhaps contribute to the observed difference in seroprevalence observed with *Anaplasma* in these regions. In the southcentral and southwestern states, increased prevalence of *Anaplasma* spp. is more likely due to *A. platys* transmitted by brown dog ticks, which are commonly present in this region [43, 44]. The use of systemic and topical acaracides may help to prevent anaplasmosis and Lyme borreliosis as both have been reported to reduce *I. scapularis* transmission of *B. burgdorferi* and *A. phagocytophilum* to dogs [45–47].

Key limitations of the present study include lack of clinical information about individual dogs that may have provided insight into veterinarians’ reasons for testing. Selective testing, which may be more common when dogs present with clinical signs characteristic of a given vector-borne disease and owner-reported travel to an area where a suspected pathogen is endemic, would be expected to create a positive bias in percent positive test rates in non-endemic areas. We addressed this concern, in part, by omitting counties reporting few test results (less than 210 over 7 years) from the maps (Figs. 1, 2, 3, 4) as we have done in both previous reports [1, 2]. Nonetheless, dogs testing positive in regions where pathogens are not known to be transmitted are most likely due to translocation of infected dogs. Large-scale relocation of dogs from heartworm-endemic areas to counties where infections are rare has been associated with an increase in local prevalence of dogs testing positive for *D. immitis* [12]. Smaller studies have documented past travel in dogs that test positive for *B. burgdorferi* in areas where disease is not known to be locally transmitted [48, 49]. Canine serologic data alone cannot confirm pathogens are established in a given area, and multiple lines of evidence, including directly testing vector mosquitoes or ticks, are necessary to confirm apparent transmission of vector-borne pathogens in new regions [1, 2, 12, 13, 47, 48]. Similarly, while the present study confirms that canine serology remains a useful predictor of where corresponding tick-borne diseases are most likely to be reported in people, limitations in both human and canine datasets suggest this finding must be interpreted with appropriate caution [2, 48–50]. Nonetheless, routine screening of dogs for vector-borne infections, as recommended by veterinary advisory groups to allow early detection of infection in individual patients [3, 4, 32], has a clear, albeit secondary, veterinary and public health benefit: careful analysis of the resulting aggregate data enhances our understanding of the changing pattern of the distribution of these vector-borne pathogens across the USA [1, 2].

## Conclusions

In this study—the third large-scale vector-borne disease serosurvey conducted since we began analyzing multi-year data sets in 2001—we have provided a comprehensive update on the frequency of positive test results for the most common canine vector-borne disease agents in the US. Several important findings were made in this extension of the study series. For the first time, the data demonstrate positive test results for each of the four vector-borne disease agents in all states. This significant finding may be explained by expanding vector range, global climate change, and increasing pet translocation, both for adoptions and for travel. Also unique to this study, the overall incidence of *B. burgdorferi*-positive results decreased relative to prior study periods, driven by the largest decreases in Lyme-endemic areas, while increases in *B. burgdorferi* were observed in non-endemic geographies, suggesting continued spread of this infection into new areas. Finally, the data show a positive association between seroprevalence of vector-borne disease agents in dogs and reported human cases of disease. Regular screening of canine pets can not only improve healthy outcomes for dogs but can also provide insight into the risk of vector-borne disease for people.

## Data Availability

Data from this study are available for review on reasonable request to IDEXX Laboratories, Inc.

## Abbreviation

ELISA: Enzyme-linked immunosorbent assay
